# A Clinical Treatise on the Endemic Fevers of the West Indies, Intended as a Guide for the Young Practitioner in Those Countries

**Published:** 1837-07

**Authors:** 


### Art. XI.

*A Clinical Treatise on the Endemic Fevers of the West Indies, intended as a Guide for the young Practitioner in those Countries.* By W. J. Evans, Esq. m.r.c.s.—*London*, 1837. Pp. 309.

Information relative to the natural history of malaria and its effects on the animal constitution, ought to possess great interest in the eyes of physicians of this country, comprising, as we believe it does, within its circuit, the colonies included, more noisome climates than are to be found within the territory of any other single power. Yet, probably because most portions of our home-territory are in general exempt from the very manifest operation of the poison, we know not any country in which the subject is more neglected than this. The compilation of Dr. Macculloch, though destined for the home-market, was not received with the attention which, diffuse and often ill reasoned as it was, it merited; and we believe that an acquaintance with the most valuable works in which the operation of malaria is traced, from those of Cleghorn and Pringle to the more recent productions of Drs. Jackson and Ferguson, is confined almost exclusively to practitioners connected with the public service. As a consequence of this when, in a season unusually moist and warm, the ordinary effects of malaria are manifested, confusion, embarrassment, and controversies among private practitioners are the result. We speak of what we have ourselves observed.

It is then with a feeling of the importance of the subject to every British practitioner, that we crave the attention of the reader to the work of Mr. Evans, the result of many years' observation of the diseases occurring in one of the most unhealthy of our colonies, St. Lucia. He is a practical writer, and, though he bestows all the attention they merit on the experiments of MM. Julia and Vauquelin on the intrinsic nature of malaria, he finds them, as others had done, inconclusive, and proceeds to the investigation in a manner more likely to elucidate the truth, by comparing the effects of the poison on the animal economy with those produced by substances such as putrescent matters, the operation of which can at any time be made the subject of direct experiment.

The first portion of the work is dedicated to a topographical description of the Island, which proves that it abounds in swamps well-suited under the action of a tropical sun for the generation of malaria. The following is his description of the mud found in one of the most virulent swamps in St. Lucia, which immediately adjoins the principal town, Castries.

"This swamp, for a foot or eighteen inches from its surface, is composed of a black mud of unpleasant smell, containing the leaves and small branches of the shrubs which grow out of it. Below this, to the depth of five or six feet, it is composed of a solid matter of a yellow colour, exactly resembling rotten horse-dung, containing some marine shells, and is principally formed of the fibrous parts of vegetables mixed up with animal matter. The odour emitted by this substance is indescribable, but disgusting in the extreme, producing a sweetish sickly taste in the mouth, pharynx, and upper part of the œsophagus, with a discharge of saline. A thermometer thrust into the sides of a canal five feet deep, rises rapidly to 100° and upwards; the temperature appears to be greater than that of any dunghill, and communicates a tingling unpleasant feeling to the hand. The vapour which arises from it is very evident, being opaque like smoke; and, though it fills the canal, ascends only to the height of four or five inches above the level of the swamp." (P. 11.)

There can be no doubt of the decomposition of vegetable and animal remains in such a situation as this; and the author, observing that swamps possess degrees of virulence proportionate to the quantity of organic matter they contain, is naturally led to retain the old opinion, which attributes the generation of malaria to the decomposition of such matter, and to reject that of Dr. Ferguson, which ascribes the origin of the poison to water during the process of drying; an opinion which we were always surprised to find so sensible a writer entertain.

We pass slightly over the author's natural history of malaria as he observed it at its sources in St. Lucia, and his very correct but by no means novel remarks on the mode of its diffusion, in order that we may give a farther account of the chapter, in which the physiological (pathological?) effects of the poison are described, regarding it as the most interesting portion of the work. In our analysis we shall aim at accuracy and condensation merely.

In the section where the effects of malaria on an *acclimated* population are examined, after giving a graphic sketch of the lymphatic or leuco-phlegmatic temperament so observable in the inhabitants of a malarious district in a temperate climate, and of that mixture of a similar temperament with great excitability of the nervous system, a combination of excessive indolence with love of pleasure, discernible in marshy regions between the tropics, he introduces some remarks on the diet to be observed in the latter situations. It is evident from his statement that persons who employ a nutritious diet, and drink a proportion of generous old wine, though subject to feverish attacks and affections of the abdominal viscera, escape the more wasting and destructive organic diseases to which the voluntarily abstemious and the poorer classes are exposed. An illustrative case is given, which will be read with interest.

The effects of malaria in a concentrated form are placed before the reader less in the shape of an abstract statement than in that of cases circumstantially detailed. A few of these we shall succinctly state. Two boatmen were hauling their boat up the beach, close to the most dangerous part of the swamp already mentioned as adjoining the Town of Castries, when they were speedily enveloped in a small cloud of vapour. One of them fell down apparently in a state of asphyxia, and the other was so affected as to be incapable of assisting him. The man most affected, after lying apparently insensible for some time, was led home, and in the course of the night was attacked with an intense ague. During the cold stage, the surface was chilled, the pulse was scarcely perceptible, and there was coma, interrupted by convulsions. In the stage of reaction the coma alternated with delirium; and there was vomiting of mucus, &c. with apparent pain, and shrinking and contortion of countenance when the epigastrium was pressed. The case was fatal in forty hours. The body was examined whilst warm; the blood was fluid, and a small quantity of troubled serum was effused between the arachnoid and pia mater; the lungs were somewhat engorged, and the stomach was intensely inflamed, containing about two or three ounces of blood. No account is given of the treatment adopted in this case, which was not under the care of Mr. Evans, so that we know not whether the plan of free bleeding during the cold stage, recommended by Dr. Mackintosh, was tried; but it would appear to us the only proceeding calculated to prevent the fatal effects of the interior congestions which were manifestly existing.

The following cases appear to us interesting as showing that those groups of external phenomena to which we give the name of diseases, how much soever they may differ among themselves, may yet have their origin in a common cause, and be but the outward and visible signs of interior conditions very closely related, if not identical with each other. The last cases, those of scarlatina, show moreover an important point of pathology, the great extent to which epidemic and contagious diseases are modified by the endemical influences of any given district. 1. Twenty-eight soldiers were employed by two planters to clear some land which was both humid and swampy. Within a week they were all brought to the hospital. Three of them died of *cholera morbus*, five of severe *dysentery*, and four of *adynamic fever*, in which the bodies became yellow and exhaled so infectious an odour that they could not be approached. The remainder had malignant intermittents, and recovered with the greatest difficulty.

2. A number of negroes were employed to get afloat a vessel which had been cast by a hurricane fifty or sixty yards from the water's edge, near the swamp already mentioned. It was to be accomplished by cutting a canal from the vessel to the *Carenage*. They abandoned their work to a man, declaring they could not bear the stench. Eighteen foreigners, principally Spaniards and Portuguese, were then employed; but the undertaking was abandoned when nearly two-thirds finished, for want of hands. The men fell ill day after day, and the greater part of them died; the first complained of faintness and vertigo, then the feet and legs became inflamed, and the swelling and inflammation quickly extended above the knee; fever came on, and death ensued in two or three days.

3. The autumnal months of 1830 were remarkable for the dry and clear state of the atmosphere, and there was little sickness in the town, excepting an epidemic of scarlet fever; it was not severe, and yielded generally to very mild treatment. By some measures connected with the erection of a church in the centre of the above-mentioned swamp, not only were several acres of marsh exposed which were before covered twice a day by the sea, and therefore to a certain extent innocuous, but a certain portion of its very bosom was laid bare. The first effect perceived was the smell which expanded itself over a considerable portion of the town; the next was a sudden change which the epidemic underwent. The efflorescence, instead of scarlet, became dusky red, occasionally approaching to purple; the tonsils, mouth, and pharynx were intensely inflamed, and of a dark colour, as if falling into gangrene; the blood drawn from the arm was of a deeper colour than in health, and the crassamentum did not separate from the serum; the pulse was small and rapid; the meninges became affected, the patient fell into a low muttering delirium, and, in a space of time seldom exceeding three days, died. After death the blood was found fluid and the gastro-intestinal mucous membrane and meninges were inflamed.

There is a short section on the effects of the poison on inferior animals. We are surprised to find the author declare in this, that at St. Lucia the only animals, except man, affected by malaria are dogs; for, in this respect, that Island must differ not only from malarious districts of the European continent, but even from some of the adjacent West Indian islands. Epizooties among oxen, horses, mules, sheep, pigs, goats, and domestic fowls, have been repeatedly observed, during the prevalence of marsh fever in the human species, in Hungary, Italy, various parts of France, and likewise, as we learn from the indefatigable and accurate Bailly,* in Guadaloupe and St. Domingo. These epizooties often appear in the form of a very destructive anthrax; and in some of them, as in the case of the dogs in St. Lucia, the gastro-intestinal mucous lining is found intensely inflamed or even gangrenous, in oxen, cows, &c. Mr. Evans assures us that the affection observed by him in dogs may be either continued or *intermittent*. M. Bailly, on the other hand, informs us, that epizooties never display any degree of intermittence, which he regards as peculiar to the human species. The former gentleman states the follow-

* Sur les Fièvres Intermittentes Simples et Pernicieuses. Par E. M. Bailly, de Blois.

ing as the symptoms of the disease in the canine race in St. Lucia: eyes dull and heavy, tongue protruded from the mouth and white, quick respiration, and sleep apparently disturbed by dreams. When the poor animal is forced to rise and seek water, he trembles in his gait and seems to reel; and finally life is terminated by a convulsion. There can be no doubt of these symptoms arising from malaria, for they can be produced at will by confining animals, recently imported, for a few days within the action of the swamp. Dogs are imported in abundance, but few survive the first year.

After relating the experiments of Gaspard, Magendie, Leuret, and Hamont, in which putrid substances were injected into the crural veins or brought into contact with the cellular or serous tissue of dogs, and death ensued with alteration of the condition of the blood, and engorgement of the lungs or some portion of the mucous membrane, Mr. Evans proceeds to relate similar experiments of his own. The results correspond with those of the French physicians, and we think ourselves justified in assuming, on the part of our readers, such an acquaintance with experiments of this kind, as to supersede the necessity of more than this reference to those of Mr. Evans. His purpose evidently was to discover whether the action of malaria was identical with that of putrid matter, thus palpably and distinctly introduced into the system. Were this identity established, it would confirm the results of a very different class of experiments, those of MM. Julia and Vauquelin, who discovered a minute quantity of animal matter in the vapour of swamps. The author, however, very properly abstains from pronouncing that the diseased conditions arising from malaria, and those produced by the experiments of himself and the French pathologists, are the same. Cases of intermittent disease, for instance, have never been produced by the introduction of putrid matter into the circulation, and only a few extreme cases of natural disease bear any resemblance to the results of such experiments. Allowance ought, however, to be made, he thinks, for the coarse nature of the experiments, and for the absence of various circumstances which modify the natural disease. We agree with Mr. Evans that the parallel he has instituted between the two sets of affections, the artificial and natural, has not as yet furnished *decisive* results; but we think he deserves credit for having diligently pursued a path of observation, which seems calculated ultimately to lead to truth.

Passing over the chapter on the influence of heat, in which the author breaks a lance with Andral on the liquefying effect which the latter attributes to it on the blood, and another with Broussais and Dr. Stevens on its agency in producing fever, and leaving the reader to consult in the volume itself eighty-eight well-reported and valuable cases, we pause on the chapter on Pathology.

We cannot say that we have derived from its perusal very distinct ideas of the author's opinion on the abstruse questions discussed in it. He appears to us to float between two doctrines irreconcilably opposed to each other, and, like other individuals in doubtful and double positions, he falls into inconsistencies. He says, for instance (at page 203): "The symptoms peculiar to the endemic fevers of the West Indies are those known under the name of bilious; thirst; vomiting either of bile or of porraceous and mucous fluids; constant nausea; agitation; anxiety; supination; frequent sighing and moaning; tenderness of the epigastrium; frontal headach; heat of skin; and quickness of the pulse. Do not these symptoms indicate a local origin, and that the stomach is the suffering organ? What is there deserving the name of *essentiality* about them, more than about those which accompany an inflammation of the pulmonary parenchyma?" Shortly afterwards, however, he remarks: "To this extent, therefore, I agree with the disciples of the Ecole Physiologique, that, where I can discover no other lesion than that of the mucous membrane of the stomach, I look upon the case and treat it as one of pure gastritis; but I differ from them in admitting the existence of a specific cause, *which*, though its *visible* effects in some cases may be confined to the gastro-enteric mucous membrane, *are* in the greater number of instances also observable either in the blood or in the nervous system, or in both." Now it does appear to us, as far as we can discern the meaning of this latter paragraph through the mist of its very faulty construction, that he admits in it the very "essentiality" which he had just before denied. There could be no reasonable objection, had Mr. Evans' opportunities of observation not enabled him to decide a controverted point of pathology, to his stating his inability to attain to a definite conclusion; but we cannot help censuring this apparent attempt at trimming and mystification.

The following is Mr. E.'s account of the lesions he discovered in twenty-six fatal cases. There was

"gastritis in every one of the twenty-six; enteritis in fourteen; the liver engorged in five; the gall-bladder inflamed in five; the kidneys inflamed, the inflammation occasionally extending through the ureters to the bladder, in three; pleuro-pneumonia in three; inflammation in the brain in three; meningitis in thirteen; change in the vital and physical constitution of the blood in eleven; inflammation of the lining membrane of the heart and large blood-vessels in two, in both of which the blood was fluid; complete *ramollissement* of the gastro-enteric mucous membrane, apparently produced by mercury, in three. Black vomit in five; in four the blood was diseased, in the other it appeared natural; in three it was accompanied with yellow suffusion. Yellow suffusion occurred in six, in all of which the blood was found changed; in three cases there was black vomit, in two the gall-bladder was found inflamed, and the orifice into the cystic duct almost impervious; in the other four the gall-bladder was healthy. Hemorrhage, blood fluid, in one." (P. 213.)

The following was the condition of the organs in eleven fatal cases, occurring in Europeans shortly after their arrival in the colony:

"gastritis in all the eleven; enteritis in six; liver engorged in one; inflammation of the gall-bladder in two; kidneys inflamed in two; spleen softened in one; œsophagus inflamed in two; softening of mucous membrane of the stomach and intestinal canal, apparently from mercury, in two; inflammation of the heart and blood-vessels in one; meningitis in four; blood diseased in one; black vomit in two; blood in the stomach in one; yellow suffusion in seven; hemorrhage into the cellular texture of the thigh, below the *fascia lata*, in one. It was in this last case that the blood was found diseased, and the spleen softened." (P. 214.)

On surveying these lists, the reader will be impressed with the fact, familiar to every observer of febrile diseases in tropical climates, that the abdominal organs are the principal seat of the local affections with which they are associated. We were not, however, prepared either by previous reading or observation, to expect that the sole invariable lesion discovered should be gastritis. With the most perfect confidence in Mr. Evans' good faith, we cannot help feeling some doubt whether his mental view of the condition of the stomach has not been in some degree troubled by the semi-Broussaism, which is manifestly his medical creed. We are the more moved to feel this doubt, because he enters into a controversy with Dr. Gilkrest for "striving to combat the opinion that yellow fever is nothing but a gastro-enteritis." This gentleman, in the article on yellow fever in the Cyclopædia of Practical Medicine, makes the very reasonable remarks, that, in the first or congested form of the disease, "the stomach is free from what is admitted by the best authorities to be evidence of inflammation; mere redness, whether in streaks in various directions, or in stellated patches of various sizes, has been on several occasions remarked in the mucous membrane, in the same degree as it is observed to occur in chronic or other diseases, or in cases of accidental death, where there is not the remotest suspicion of gastritis. Spots of a purple colour are much more common than those of a bright red; a perfectly pale state of the membrane is far from being of a rare occurrence." We cannot help thinking that, had not Mr. Evans' mind received a bias in favour of a gastric theory of endemic fever, he would have acquiesced in the reasonableness of these observations of Dr. Gilkrest, rather than controverted them; and a perusal of his own description of the appearance of the stomach, detailed and graphic as it is, has not convinced us that the redness he describes has not in some instances been the result of passive congestion, even during the agony. He places too much reliance on mere vascularity and likewise on mucous secretion as evidences of gastric inflammation. (See our notice of Dr. Yelloly's paper, *ante* p. 138.)

Mr. Evans' practical precepts for the treatment of the diseases of the West Indies display a discrimination of the circumstances demanding the employment of powerful remedies which is creditable to his judgment. Had his rule for resorting to bleeding in the cold stage of intermittent been more nearly approximated to than it has been, we think that this remedy would have ranked higher in public esteem than it does. He considers a degree of congestion of internal organs inseparable from the cold stage of an intermittent, and does not resort to bloodletting when such affection may be considered as existing only to the natural extent. "Should," however, "the patient become apoplectic, or should the stupor be profound and accompanied with convulsions; should the difficulty of breathing be considerable and threaten suffocation, or be performed only in a sitting posture; should the sub-crepitating *râle* be diffused over both lungs, and the sound on percussion be duller than usual, we must resort to prompt and energetic measures; to bleeding from the arm to an extent proportionate to the urgency of the symptoms and the strength of the constitution, to cupping near the organ affected, &c." We agree with the author that bloodletting is not requisite in an ordinary cold stage; but we think that degrees of cerebral and pulmonary congestion much slighter than he has described would demand this measure.

We do not deem it necessary to transfer to our pages the author's opinion regarding the treatment of every form of the endemic fever he observed, our wish being that such of our readers as either at present feel, or are likely to feel, the subject one of direct interest, should consult the work itself. Our own experience of the diseases of warm climates leads us to acknowledge the general prudence and sagacity of his practical precepts. Were we disposed to express any doubt or dissent, it would be in the case of what he calls continued inflammatory fever, which he says has been termed gastritis, gastro-enteritis, gastro-meningitis, gastro-enterite *inflammatoire* (evidently a redundancy), inflammatory yellow fever, &c. In this disease, he says, "We bleed largely, to the extent of thirty ounces, more or less, according to the constitution of the patient; we place him in a tepid bath, and return in a couple of hours. If the patient has vomited during the interval, if there remains the slightest pain on pressing the epigastrium, if the head be not relieved, or if there be any sense of heat or burning in the stomach, we repeat our bleeding: we then apply leeches to the epigastrium should they be necessary; but in general if any gastric affection remain after the second bleeding towards the close of the first twenty-four hours, we repeat it a third time, and apply the leeches afterwards." He subsequently says, "the disease may continue either in consequence of our efforts not having been sufficiently energetic, or because we have combined them with others which have counteracted the good effect which we might have otherwise obtained. I mean calomel and purgatives. The British practitioner is haunted with these medicines." We must of course speak with diffidence of the disease the author observed at St. Lucia; but if such rules are intended to be applied to yellow fever in general, we must be allowed to intimate to him that his method, both as to the amount of bloodletting and his total abstinence from calomel, is at variance with that suggested by the best authorities on this subject, British and American, unquestionably the most experienced and energetic practitioners who have dealt with this disease. We are perfectly ready to admit that calomel and purgatives have been abused by certain physicians; but, on the other hand, we have seen abstinence from these measures converted into a much greater abuse; and have witnessed more misery and waste of life from leeches, gum-water, and costive bowels, than from any degree of purgation. How long will medical men persist on thinking that, if any given proceeding be wrong, its directly opposite must necessarily be right?

We now take leave of our author by recommending his work to general perusal, thinking it a valuable one, though we have not hesitated to offer occasional (we trust candid) comments on certain points of doctrine and practice it contains, which we consider of a questionable nature. We think his opinions have received a tinge from long residence among French colonists, and must take the liberty of intimating to him that his style has not escaped the infection, for there are several *gallicisms* in it. The word "report," for instance, occurs more than once in the sense of relation or connexion between objects, a meaning which the French "rapport" has, but which the English word has never yet borne. Perhaps, by way of compensation, his French is occasionally *anglicised*, for (page 304) we have "inflammatorie" for "inflammatoire."

Mr. Evans's, however, is a very good book; and, when compared with *all* the books on the subject of West Indian fever, which were in existence some thirty years since, when we had particular occasion to search for such works, it affords a most gratifying proof of the great progress of medical science in modern times.

